# Capacity analysis of short-distance continuous diversion areas on mountainous urban expressways

**DOI:** 10.1371/journal.pone.0306881

**Published:** 2024-10-09

**Authors:** Xiaoyu Cai, Ling Jin, Bo Peng, Yongqi Li, Yang Chen

**Affiliations:** 1 College of Smart City, Chongqing Jiaotong University, Chongqing, P. R. China; 2 Key Laboratory of Spatio-Temporal Information in Mountainous Urban Areas, Chongqing Municipality, Chongqing, P. R. China; 3 College of Traffic & Transportation, Chongqing Jiaotong University, Chongqing, P.R. China; Nanjing Forestry University, CHINA

## Abstract

The short-distance continuous diversion area plays a crucial role within mountainous urban expressway systems, significantly enhancing the efficiency of specialized road sections through capacity analysis. This study develops a capacity calculation model tailored to the diversion area’s unique characteristics and principal capacity-influencing factors. Initially, the research focuses on a specific short-distance continuous diversion area of a mountainous urban expressway, employing video trajectory tracking technology to gather trajectory data. This data serves as the basis for analyzing road and traffic characteristics. Subsequently, the model computes the capacity influenced by eight variables, including diversion point spacing and deceleration lane length, using VISSIM simulation experiments. A gray correlation analysis identifies key factors, which guide the establishment of the model’s fundamental structure through two-factor surface fitting results. Mathematical statistical methods are then applied to resolve the model’s parameters, culminating in a robust capacity calculation model. The findings reveal that diversion point spacing, along with primary and secondary diversion ratios, significantly influence capacity. Notably, the capacity exhibits a marked quadratic polynomial relationship with the primary diversion ratio and diversion point spacing, and a linear relationship with the secondary diversion ratio. The model’s validity is confirmed through a case study at the diversion area north of Huacun Interchange in Chongqing Municipality, where the discrepancy between calculated and actual capacities is under 5%, underscoring the model’s high accuracy. These results offer valuable theoretical and methodological support for the planning, design, and traffic management of diversion areas.

## 1. Introduction

Urban expressway systems are being widely promoted and implemented to connect major functional areas and urban clusters while alleviating urban traffic congestion [1]. Mountainous urban expressways are limited by factors such as topography and geomorphology, and diversion ramps are usually set up consecutively over short distances [2]. Vehicles in such diversion zones frequently experience lane changes, deceleration diversions, and intertwining conflicts [3], which can easily lead to traffic congestion and result in the inability of the expressway to properly perform its function [4]. Therefore, it is of great significance to analyze the operation mechanism of the diversion area, investigate the influencing factors, construct a capacity calculation model, and accurately calculate the actual capacity of the diversion area to ensure the efficient operation of vehicles and realize the functional advantages of expressways.

This paper focuses on exploring the short-distance continuous diversion areas of mountainous urban expressways. It conducts a thorough analysis of road and traffic characteristics to pinpoint the key factors affecting traffic capacity. By employing a two-factor surface fitting approach, it establishes a clear understanding of the relationship between each factor and traffic capacity. The development of a robust traffic capacity calculation model greatly contributes to advancing research in traffic capacity modeling within short-distance continuous diversion areas.

## 2. Literature review

The diversion zone is an important part of an urban expressway system [5], and the study of its capacity is of great significance for the optimization of urban traffic management and planning. To analyse the capacity more objectively and systematically, scholars have carried out a large amount of research on the characteristics of the influence zone, the factors influencing the capacity, and the capacity calculation model.

### 2.1 Influence area characteristics

Many studies have been conducted on the characteristics of the common types of ramps connecting segments and their influence areas, and researchers have mainly focused on expressway ramps [6], ramp-influenced diverging and merging areas [7], and intertwining areas [8]. Traffic operation characteristics and complex traffic flow studies have also been conducted [9, 10]. Tian [11] employed a continuous cellular automaton (CCA) model incorporating entrance and exit ramps to delve into the intricate traffic flow and driving behaviours on expressway ramps. Gong [12] scrutinized the operational characteristics of ramp diversion zones, analyzed two exit ramp diversion areas under different lane operation strategies, and examined the distribution of vehicle types across lanes.

### 2.2 Influencing factors

Capacity is affected by a variety of factors, including roadway conditions, traffic conditions, and other factors, and most existing studies analyzed single or partial influencing factors for roadway and traffic conditions [13]. In terms of roadway conditions, Pompigna [14] found that the number of mainline lanes and road characteristics have a greater impact on capacity, and Zheng [15] concluded that the ramp type, number of lanes, and concourse length are the main factors affecting capacity. Ru [16] conducted research on urban expressways and found that lane width, lane position, number of lanes, and main segment length have significant impacts on the traffic capacity of urban expressways. Regarding traffic conditions, Calvi [17] found that the capacity of single-lane exit ramps was significantly reduced when vehicles were traveling at low speeds. Asgharzadeh [18] found that vehicles traveling in the merging area were affected by a variety of factors, such as mainline traffic volumes, speeds, and roadway alignment. Li [19] investigated the traffic characteristics and vehicle behaviour within deceleration lanes in diversion zones, revealing that vehicles traveling in deceleration lanes are influenced by various factors including mainline traffic volume, speed, and road alignment.

### 2.3 Traffic capacity calculation model

Many scholars have conducted in-depth research on the calculation model of the capacity of expressway diversion and merging areas, which mainly includes three types of methods: actual data estimation, simulation modeling analyses, and theoretical derivation analyses.

Actual data estimation is the most commonly used method in the field of traffic engineering. Scholars typically analyse a specific impact area or instance within a certain region and then provide recommended values or charts, often in conjunction with the Highway Capacity Manual [20]. Later, some scholars [21] established a general model for capacity estimation based on actual data, and this direct estimation method replaced the complicated query form in HCM2000 and was included in HCM2010 [22]. However, the method of estimation through actual data is often affected by a variety of factors such as weather and accidents when collecting data, which has certain limitations.

With the development of computer technology, micro-simulation models have gradually been used to analyse accessibility because they can simulate traffic flow changes over long periods. Xie [23] utilized the Federal Highway Administration’s Traffic Software Integration System (TSIS) to analyse the operational capacity of single-lane exit ramps and parallel single-lane exit ramps. However, high-precision micro-simulation usually requires high computational resources, especially when simulating large-scale road networks or long-time simulations, which require more powerful computer or server support.

To enhance the extensiveness and applicability of the model, theoretical analysis methods are proposed to establish a capacity calculation model by analyzing the operation mechanism in the influence zone. Some scholars mainly focus on the traveling patterns of vehicles in the influence zone through gap acceptance theory models [24]. Wang [25] calculated a model for the length of the connection section between ramps and at-grade intersections established based on lane-changing theory, acceptable gap theory, traffic fluctuation, and the Webster vehicle delay model. Shen [26] built a computational model for the merging area under different types of traffic flow, taking into account individual differences in both randomly arriving vehicles and driving behaviours, based on the gap acceptance theory. However, gap acceptance theory models are usually based on certain assumptions that differ from the complexity of the actual traffic flow and affect the accuracy of the models. There is also some existing research that has proposed methods based on traffic flow theory to establish a capacity calculation model by comprehensively evaluating the state of traffic flow in the impact area. Chen [27] developed a computational model for the weaving, merging, and diverging capacities of dedicated lanes within the central median barrier of expressways, building upon an analysis of weaving behaviour at interchanges with central median barriers on urban expressways. Fu [28] employed the Greenshields traffic flow model to assess the differences in lane capacities between tunnel segments and adjacent regular road segments through lane calibration, thereby evaluating the expected lane capacity of expressway tunnel segments. The traffic flow theory-based methods account for the stochastic and cyclical characteristics of traffic flow and can better adapt to the actual situation.

### 2.4 Previous research summary

Although scholars domestically and abroad have conducted a certain degree of in-depth research on the capacity of urban expressway diversion areas, there are still some defects, including the following:

- The characterization of continuous diversion areas over short distances is weak, especially for mountainous cities with complex road conditions.
- Most existing studies analyse individuals to some of the influencing factors; there are few studies that comprehensively consider the impacts of various combinations of factors on capacity.
- The existing research results most use ramps and diversion and merging areas to establish capacity calculation models, and they are not applicable to the short-distance continuous diversion areas on mountainous urban expressways. A need exists to construct the capacity calculation model specifically for such characteristics.

To solve the above problems, this study models the capacity in a more accurate way under mixed traffic conditions based on characteristic analyses and multifactor combination analyses. The research motivation and contributions of this paper are as follows:

- Study the characteristics of roads and traffic in diversion areas based on empirical data for mountainous cities, exploring the spatial distribution characteristics and variations of traffic volume, diversion ratio, and vehicle speed.
- Based on simulation experiments, capacity under various combinations of influencing factors is obtained. The principal factors affecting capacity are determined using gray correlation analysis, followed by multifactorial combination analysis of the identified influencing factors.
- Using theoretical derivation and analysis methods to construct a capacity model for short-distance diversion areas, we describe the laws of traffic flow operation at the macro and micro levels and perform a quantitative assessment of the capacity.

The rest of the paper is organized as follows. "Data" describes the process of data acquisition and processing, and "Methods" analyses the road characteristics and traffic characteristics, and explains the process and results of capacity modeling. "Results and Conclusion" explains the process and results of capacity modeling, conclusions, recommendations, and outlook.

## 3. Data

### 3.1 Video data collection

**Fig 1:**
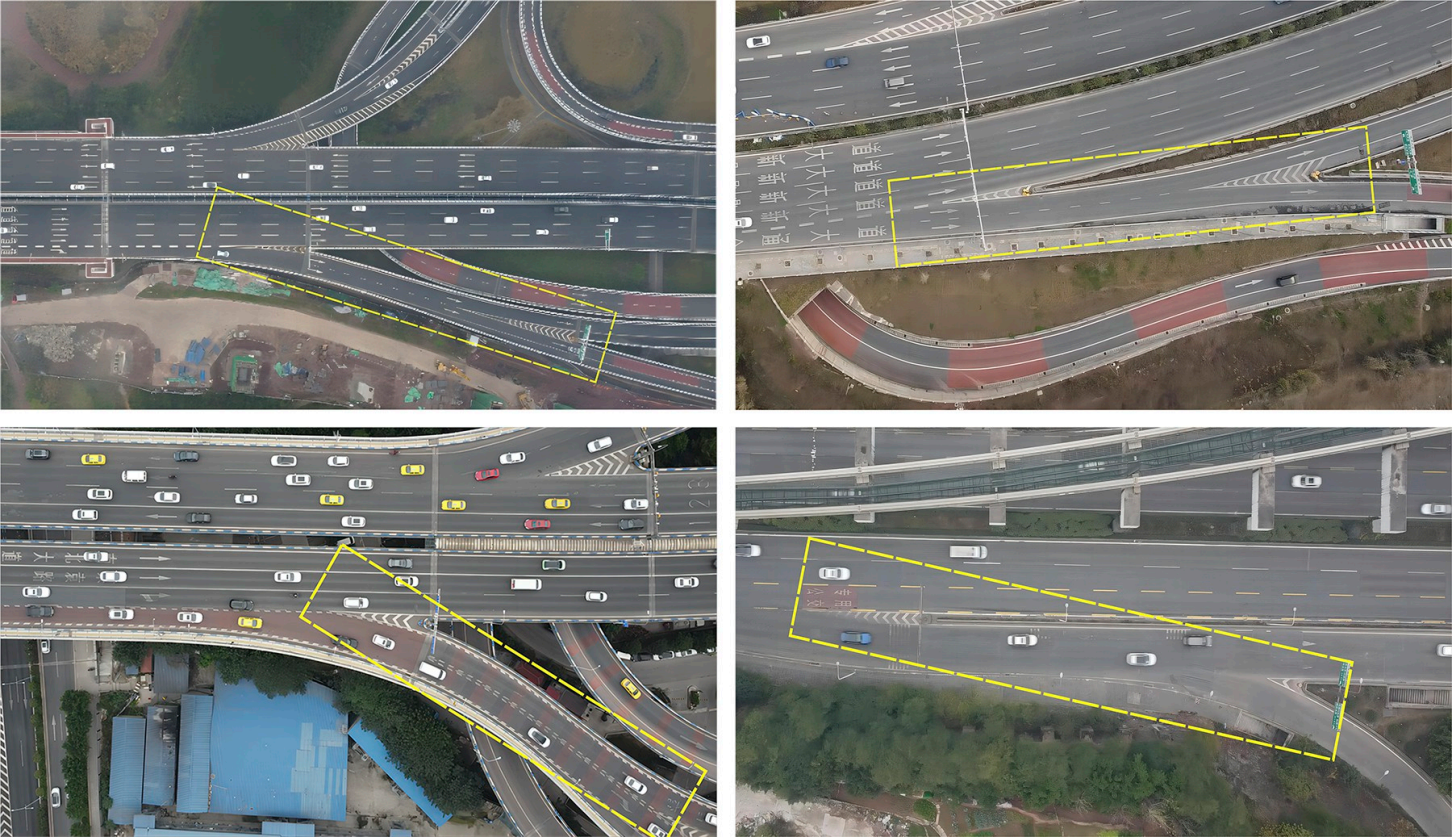
Survey area: (a) West of Libai Interchange; (b) East of Libai Interchange; (c) Huacun Interchange; and (d) Xinhua Interchange.

To collect traffic flow data and analyse the characteristics of the diversion areas of mountainous urban expressways, the Libai Interchange in Yubei District, Huacun Interchange in Yuzhong District, and Xinhua Interchange in Dadukou District of Chongqing Municipality, China, which have typical short-distance continuous diversion characteristics, are selected as the investigation sites. Fig 1 shows the locations of typical short-distance continuous diversion areas in specific investigation areas.

The geometric parameters of the four diversion areas were gathered, including the spacing between diversion points, the length of deceleration lanes, and the number of lanes. Additionally, to explore the traffic characteristics of the short-distance continuous diversion area, a traffic survey was conducted at the west diversion area of the Li-Bai Interchange in Chongqing during off-peak hours (15:00–17:00) and peak hours (7:00–9:00, 17:00–19:00) on weekdays. Parameters assessed during the survey included traffic volume and traveling speed. To fulfill the research objectives and data requirements, aerial videography using a drone (DJI MINI 2 model) was employed for video data acquisition. This drone boasts a flight duration of 30 minutes, a maximum flight altitude of 500 meters, and supports crisp 4K ultra-high-definition video transmission in real-time to the control interface, thus meeting the research demands of this study.

### 3.2 Video data processing

#### 3.2.1 Data extraction

**Fig 2:**
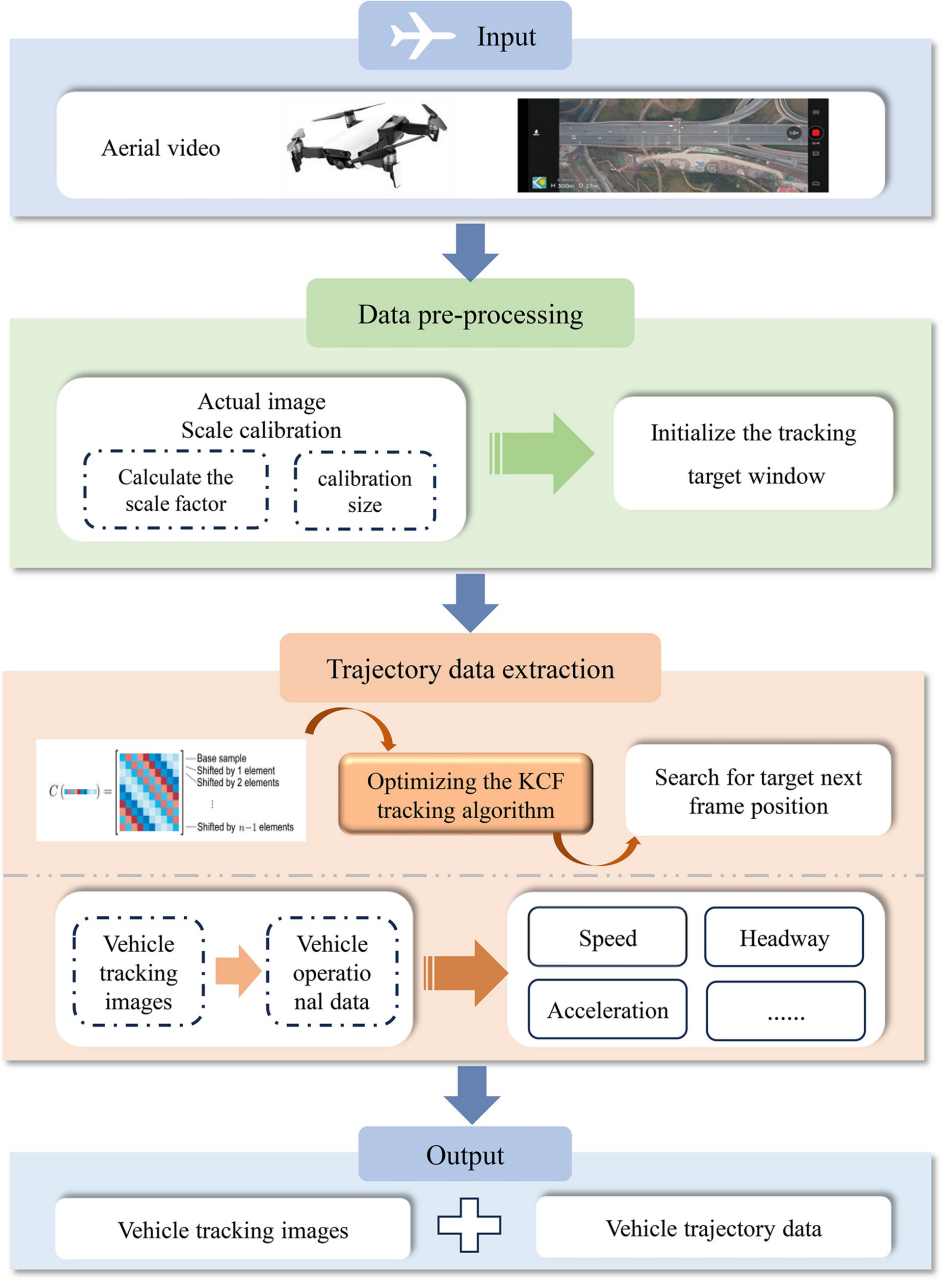
Flowchart of trajectory data extraction.

To study the microscopic driving behaviour of vehicles in the diversion area, this study computationally acquires traffic flow data by extracting vehicle trajectory coordinates from aerial video. Using a vehicle identification tracking algorithm, the position of the target vehicle is searched on each frame of a video image, and its coordinate data is output. Then, the actual ratio of the image within the study area (the ratio between the actual length and the number of pixel points in m/pixel) is calibrated. Finally, the traffic flow data are obtained via calculation. Fig 2 shows the main flow of the data extraction.

Using the unmanned aerial vehicle (UAV) video obtained from the survey with a vehicle identification and tracking algorithm, the number and coordinate data of the vehicle are output as shown in Table 1. The frame rate of the video acquired in this study is 30 frames/s, and the interval of data storage is set to 15 frames, i.e., coordinate data are acquired once every 0.5 s.

**Table 1:** Vehicle location information data.

Current frame	Vehicle number	Upper left X	Upper left corner Y	Rectangular width	Rectangular height	Center Coordinate X	Center Coordinate Y
30	1	1404	679	41	26	1424.5	692
30	2	1136	651	43	18	1157.5	660
30	3	853	643	38	16	872	651
…	…	…	…	…	…	…	…
30	12	490	560	47	19	513.5	569.5
30	13	1535	537	40	17	1555	545.5
30	14	1373	535	46	24	1396	547
30	15	1072	531	45	20	1094.5	541

The actual scale of the image is calibrated, and, based on the frame interval, vehicle number, and coordinate data, information such as the vehicle traveling distance, speed, and headspace can be derived via calculation. The specific calculation formulas are as follows.

Vehicle travel distance:

$$D=\sqrt{{\left(X_{i+1}-X_{i}\right)}^{2}+{\left(Y_{i+1}-Y_{i}\right)}^{2}}\times w$$ (1)

Where *D* represents the distance traveled by the vehicle in m, *X*_*i*_ represents the horizontal coordinates of the center of the vehicle in the *i*th frame in pixels; *Y*_*i*_ represents the *i*th frame vehicle center vertical coordinate in pixels, *w* represents the actual scale of the image in m/pixel.

Vehicle speed:

v=D/T (2)

where: *v* represents the vehicle speed in m/s, *D* represents the distance traveled by the vehicle in m, and *T* represents the time interval in s.

Distance from the vehicle in front:

$$d_{x_{j}}=(X_{j-1}-X_{j}-L_{j})\times w$$ (3)

where: $d_{x_{j}}$ represents the distance between the *j*th vehicle and the front vehicle in m, *X*_*j*_ represents the *j*th horizontal coordinates of the upper left corner of the vehicle rectangle in pixels, *L*_*j*_ represents the length of the *j*th vehicle in pixels, and *w* represents the actual image scale in m/pixel.

From the above formula, the vehicle operation data are as follows in Table 2.

**Table 2:** Vehicle operation data.

Current frame	Vehicle number	X Distance (pixels)	Y Distance (pixels)	Total Distance (pixels)	Speed (km/h)	Speed angle (°)	Distance from the vehicle in front (m)	Acceleration (m/s2)
30	1	60.0	11.0	61.0	66.4	- 10.4	0.00	0.20
30	2	61.0	4.0	61.1	66.6	-3.8	34.06	1.27
…	…	…	…	…	…	…	…	…
60	1	57.0	14.0	58.7	63.9	- 13.8	0.00	- 1.18
60	2	59.0	7.0	59.4	64.7	-6.8	33.76	0.12
…	…	…	…	…	…	…	…	…
60	14	81.0	1.0	81.0	88.2	-0.7	16.50	0.00
60	15	83.0	0.0	83.0	90.4	0.0	38.00	-0.60

#### 3.2.2 Accuracy test

**Fig 3:**
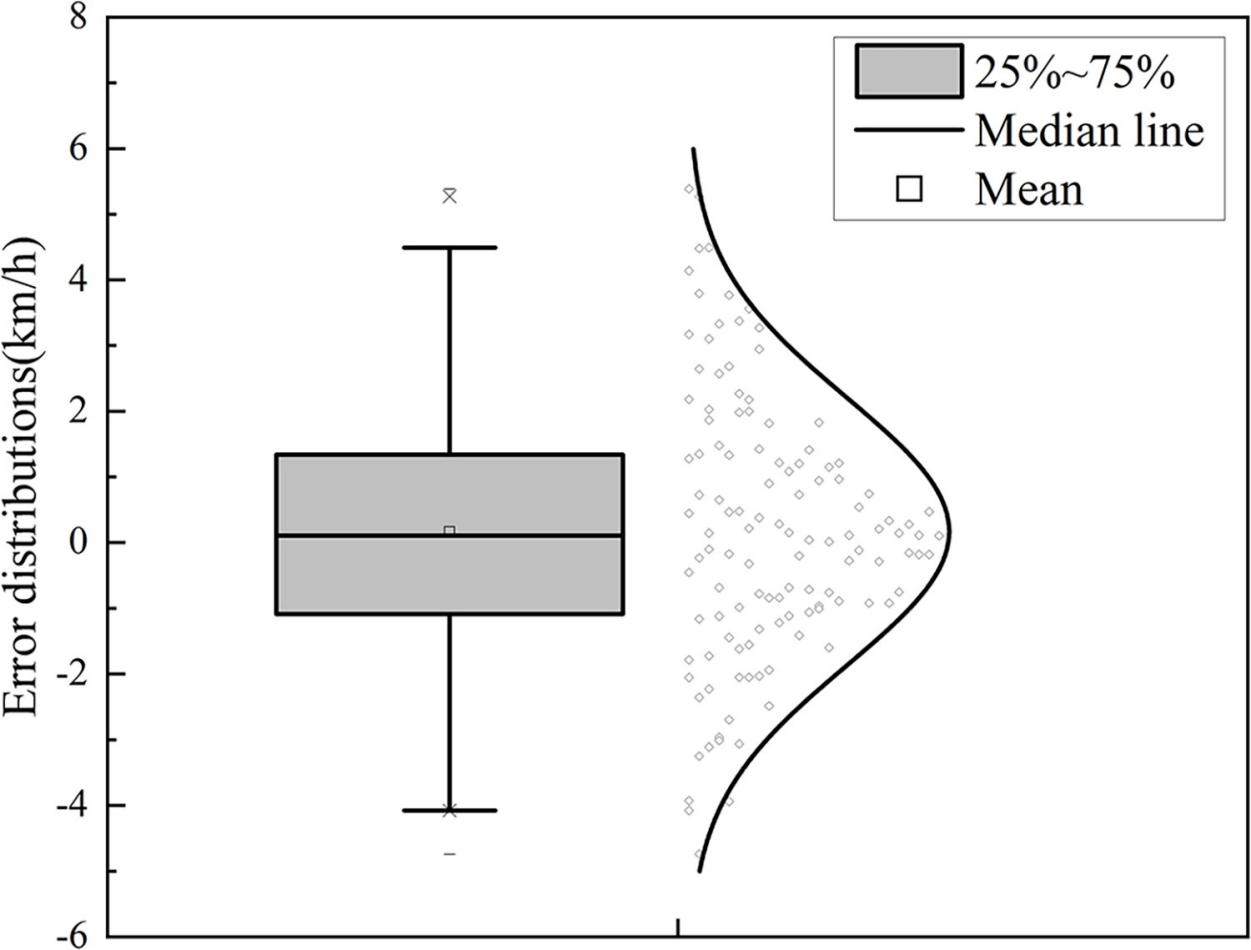
Speed detection data comparison.

**Fig 4:**
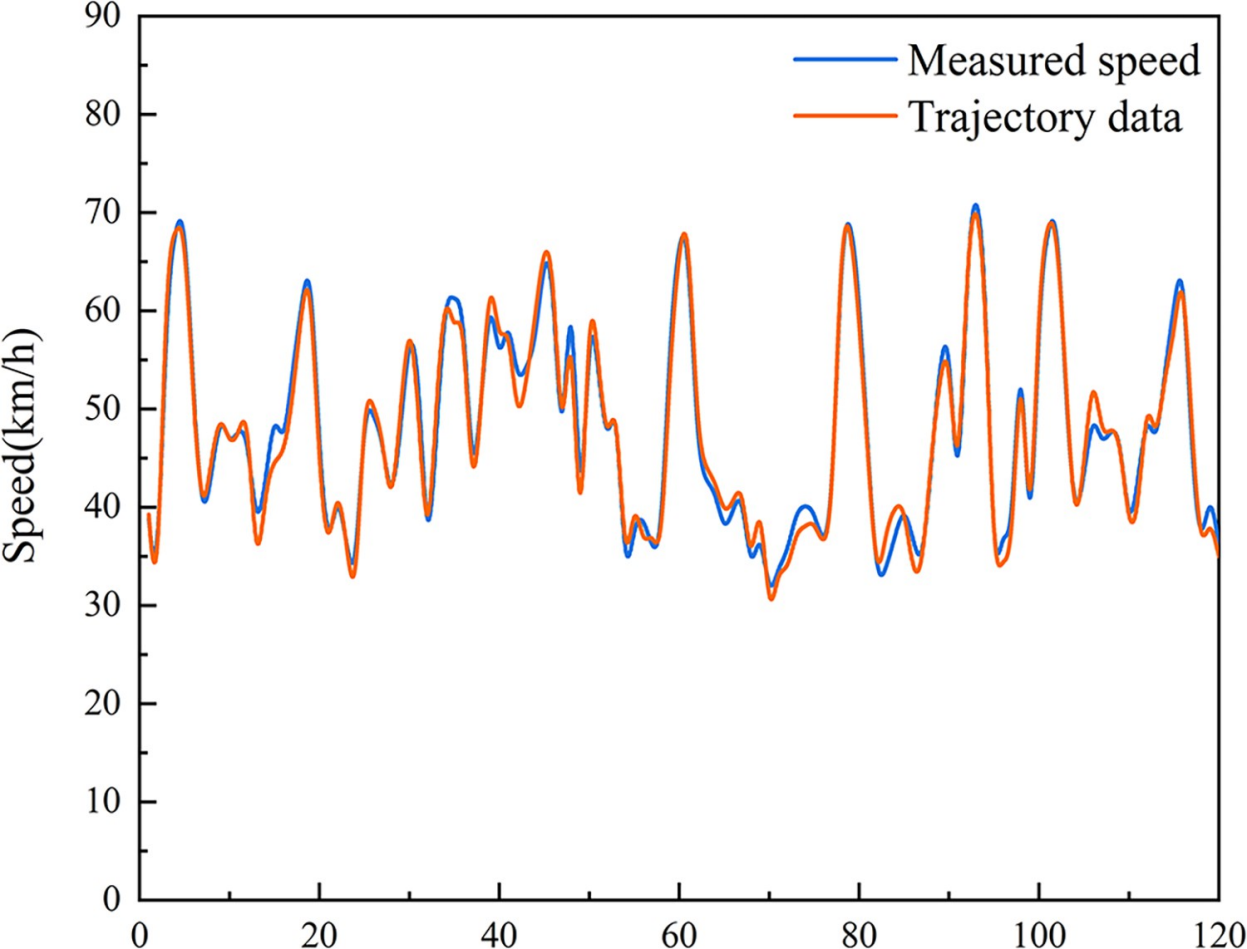
Distribution of speed error.

To test whether the trajectory data extraction method used in this paper is accurate and effective, the vehicle traveling speed was selected as the evaluation index with which to test the accuracy of the trajectory data. In this experiment, the Li-Bai interchange diversion area was selected as the test site, and the comparison results of the trajectory data and the measured speed are as shown in Fig 3. The extracted speed is closer to the real value. The error distributions of the two are shown in Fig 4 As. The error is basically normally distributed with an average value of 0.21km/h, and the maximum absolute value of the error is less than 5km/h. The method of extracting the vehicle trajectory data has high accuracy.

## 4. Methods

**Fig 5:**
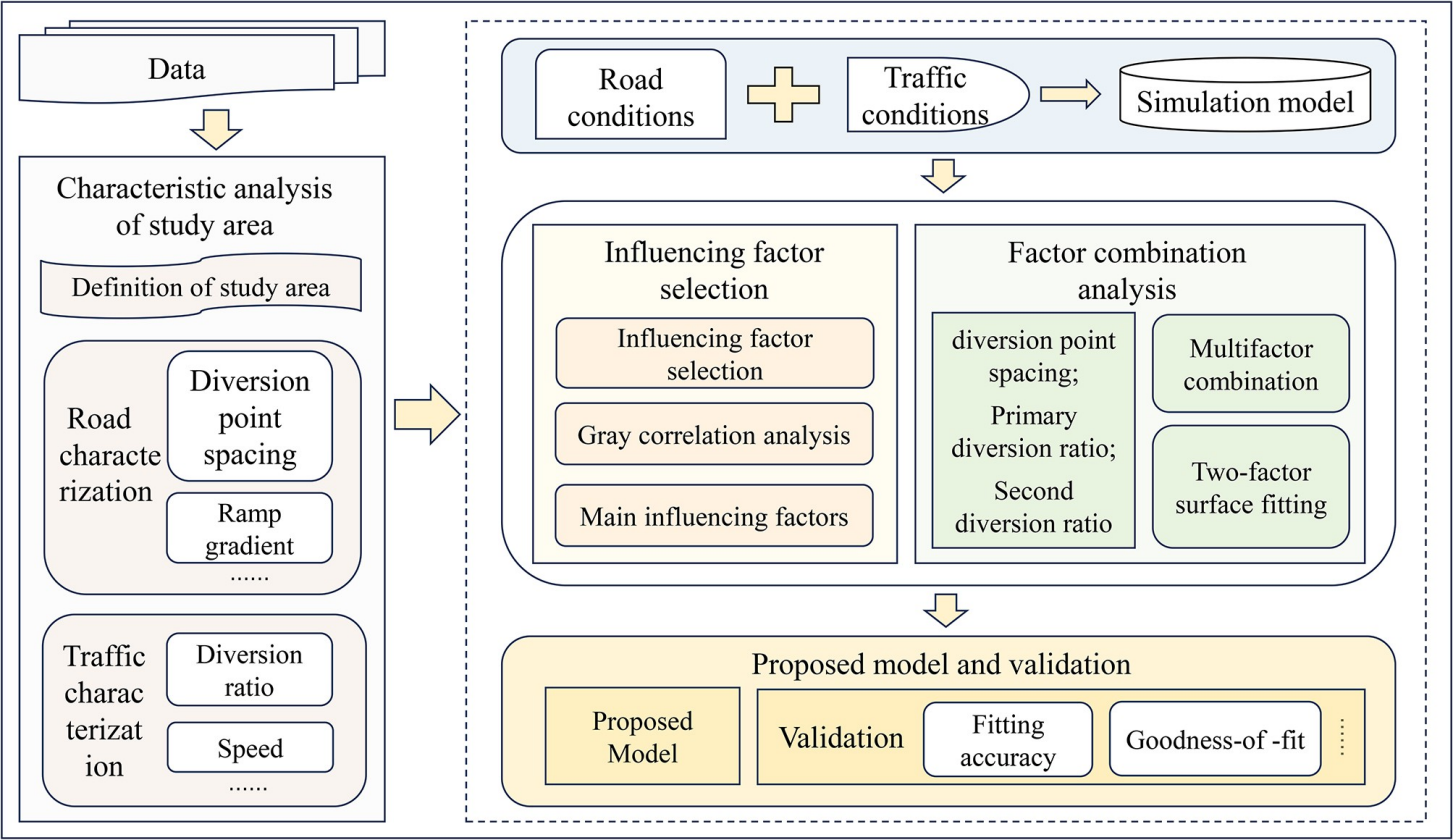
Framework of methodology.

A ’Characteristic Analysis—Model Construction’ approach was employed in this study. Initially, the ’Characteristic Analysis’ segment delineated the study area and analyzed both road and traffic characteristics using empirical data. Subsequently, the ’Model Construction’ segment involved three components: constructing a simulation model, analysing combinations of major influencing factors, and building and validating the computational model. The main framework is illustrated in Fig 5.

### 4.1 Analysis of study area characteristics

#### 4.1.1 Definition of study area

**Fig 6:**
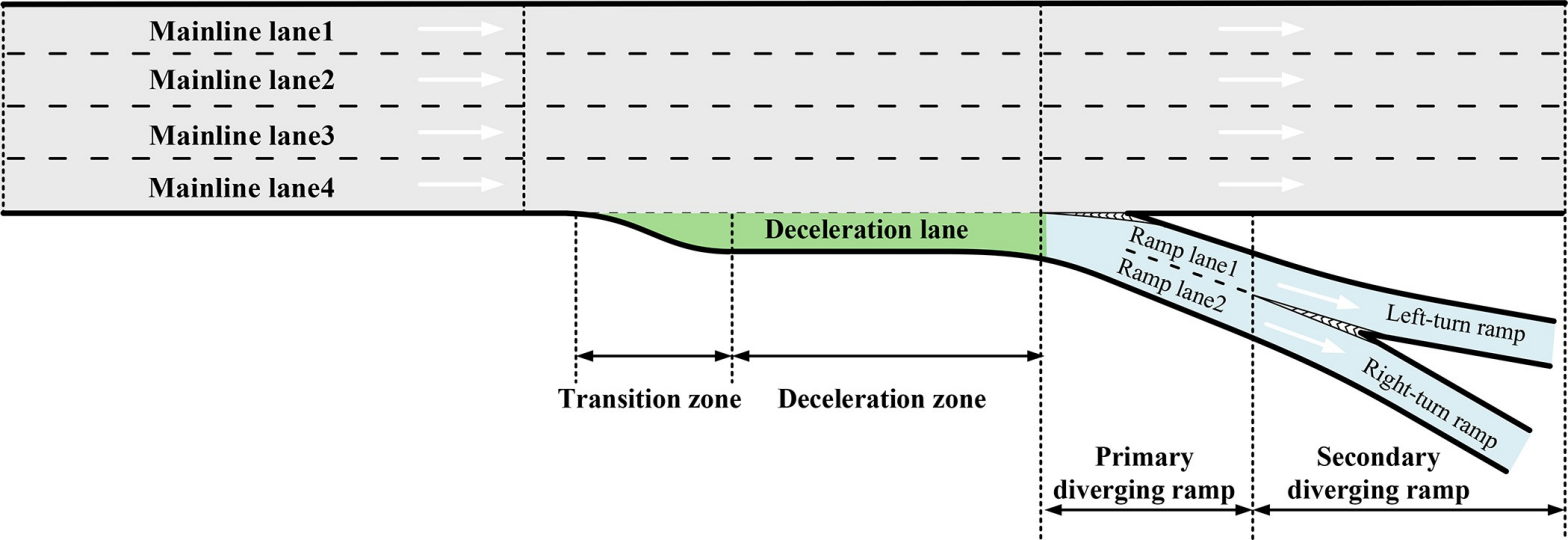
Schematic of short continuous diversion zones for urban expressways in mountainous areas.

Referring to China’s "Highway Stereo Intersection Rules" [29] and other relevant standards and specifications, the short-distance continuous shunt area is defined as an area with two or more consecutive separations within a specified range where the distance between the shunts is less than 120 m. This paper focuses on the ’right ramp continuous shunt’ area, prevalent in mountainous urban expressway systems, as illustrated in Fig 6. A survey of 91 continuous diversion areas in the mountainous urban region of Chongqing showed that approximately 30% of the secondary diversion ramps were spaced less than the minimum required distance of 120 m. Such proximity poses challenges due to the frequent need for lane changes within the diversion area. Excessively short distances between continuous diversion points can increase interference and conflicts between the mainline and ramps, consequently diminishing service levels and compromising safety standards.

#### 4.1.2 Characteristic analysis

Combined with the analysis of field survey data and domestic and international studies, this research summarises the characteristics of short-distance continuous diversion areas from both road and traffic perspectives.

1. *Road characterisation*. Due to the limitations of terrain conditions, the mountainous urban expressway diversion point spacing is generally shorter [30]. and the length of the deceleration lane is generally less than the standardized design value of 130 meters. The overall line technical standards are lower, the ramp gradient is more than the limit value of 8%, and the slope is larger. However, spiral interchanges and other special forms also exist. The ramp curve radius is less than the limit of the minimum radius of 50 meters and is not conducive to traffic safety. The number of lanes on the main line upstream and downstream is generally three or four lanes, the primary diversion ramps are mostly two or three lanes, and the secondary diversion ramps are mostly two lanes or a single lane. The lanes are in good condition.
2. *Traffic characterisation*. Traffic in short-distance consecutive diversion areas is influenced by all lanes on the mainline. When traffic conditions are good and lane changes are not required, drivers tend to use the middle mainline lanes, with the innermost two lanes having the most stable traffic volumes and being least affected by diversion behaviour. Conversely, the two outermost lanes are the most affected by diversions, with significant variations in traffic volume. In the downstream area of secondary diversion points, traffic volume in each lane generally remains stable.

The diversion ratio, derived from traffic data and presented in Table 3, indicates that under free-flow conditions, the majority of vehicles access the ramp through the deceleration lane, achieving a diversion ratio of 0.378 with minimal impact on the mainline lanes. In contrast, during congested periods, reduced mobility in the deceleration lane inhibits lane changes, compelling some vehicles to access the ramp via mainline lane 4. This results in a diversion ratio of 0.428, substantially affecting traffic flow.

**Table 3:** Lane diversion ratio.

Video	Operating Conditions	Total Diversion Rate (veh/h)	Exit Flow Rate (veh/h)		Diversion Ratio		
			Main Lane 4	Main Lane 5	Main Lane 4	Main Lane 5	Total Proportion
1	Free-Flow	3292	96	1148	0.029	0.349	0.378
2	Free-Flow	3216	104	1052	0.032	0.327	0.359
3	Congested	3624	316	1208	0.087	0.333	0.421
4	Congested	3580	340	1192	0.095	0.333	0.428

Table 4 presents lane speed characteristics and variations within the diversion area. Under free-flow conditions, high speeds facilitate stable vehicle operation, decreasing from lane L1 to L4 accompanied by increasing speed dispersion. L1 maintains higher, more consistent speeds with minimal impact from diversions, while L4 shows significant reductions and variability due to traffic and vehicles slowing for the exit ramp. During congestion, speeds decrease across all lanes, resulting in chaotic vehicle operations and pronounced speed disparities. This pattern of speed reduction from L1 to L4, with enhanced variability, continues; particularly in L4, speeds significantly drop from 58.91 km/h to 21.35 km/h, highlighting its considerable susceptibility to diversions.

**Table 4:** Diversion area lane speed characteristics.

Lane Designation	Number	Free-Flow (km/h)		Free-Flow (km/h)	
		Mean Value	Standard Deviation	Mean Value	Standard Deviation
Main Lane 1	L1	83.36	7.04	71.13	10.27
Main Lane 2	L2	76.99	7.75	66.02	12.74
Main Lane 3	L3	75.77	8.40	62.57	13.92
Main Lane 4	L4	58.91	10.14	21.35	14.13
Deceleration Lane	L5	53.64	8.20	19.81	10.96
Main Lane 1	L6	50.74	9.24	25.86	14.45
Main Lane 2	L7	47.31	7.96	20.96	10.83
Left Turn Ramp	L8	45.78	9.14	38.86	13.15
Right Turn Ramp	L9	41.42	7.42	23.90	10.02

Vehicles in short-distance diversion areas frequently change lanes owing to spatial and temporal constraints, which complicates traffic dynamics and reduces throughput. Table 5 illustrates the significance of analyzing these behaviors. Lane-changing rates for inner mainline lanes 1 and 2, and ramp lane 2, consistently stay below 0.3, reflecting minimal impact from merging traffic. Notably, L4 records the highest rate at nearly 0.9, serving all turning vehicles. Lanes L3 and L6 exhibit variable rates: L3’s rate is 0.428 in free-flow conditions and decreases to 0.139 during congestion, while L6’s rate escalates from 0.417 to 0.958 under congested conditions, attributed to mandatory lane changes caused by queuing.

**Table 5:** Lane-changing rates.

Lane Designation	Number	Number of Lane-Changing Vehicles (veh)		Total Number of Vehicles (veh)		Lane-Changing Rate	
		Free-Flow	Congested	Free-Flow	Congested	Free-Flow	Congested
Main Lane 1	L1	33	56	192	199	0.172	0.281
Main Lane 2	L2	10	40	222	245	0.045	0.163
Main Lane 3	L3	89	22	208	158	0.428	0.139
Main Lane 4	L4	179	263	201	293	0.891	0.898
Main Lane 1	L6	10	23	24	24	0.417	0.958
Main Lane 2	L7	14	52	287	357	0.049	0.146

### 4.2 Simulation model establishment and verification

A simulation model was developed based on comprehensive surveys of road and traffic conditions within the diversion area adjacent to the Li-Bai Interchange. This model includes parameters such as traffic volume, vehicle composition, desired velocity, and diversion ratio. Four distinct vehicle paths were delineated based on real-world conditions, with precise control over input parameters for each lane to accurately replicate the operational dynamics of the diversion area under various traffic scenarios.

A sensitivity analysis was performed on these parameters using a single-variable control method, with dual metrics serving as evaluation criteria. The analysis revealed a hierarchical relationship among the parameters, with desired velocity as the most influential, followed by headway (CC1), subsequent variation (CC2), safety distance reduction coefficient, and stopping gap (CC0). Other parameters showed marginal effects on the simulation model, with discrepancies below 5%. Thus, the pivotal influencing factors in the simulation model for the short-distance continuous diversion area were identified as desired velocity, headway (CC1), subsequent variation (CC2), safety distance reduction coefficient, and stopping gap (CC0). Calibration of these parameters was conducted using trajectory data from aerial video surveys, which captured vehicle movements under unimpeded conditions within the diversion area. Notable calibrations included setting the stopping gap at 2.37 m, headway at 1.41 s, and subsequent variation at 3.27 m. Key parameters within the Wiedemann99 model were adjusted accordingly: CC1 to 1.41 s, CC0 to 2.37 m, CC2 to 3.27 m, the safety distance reduction coefficient to 0.2, CC4 to -0.95, and CC5 to 0.95.

**Fig 7:**
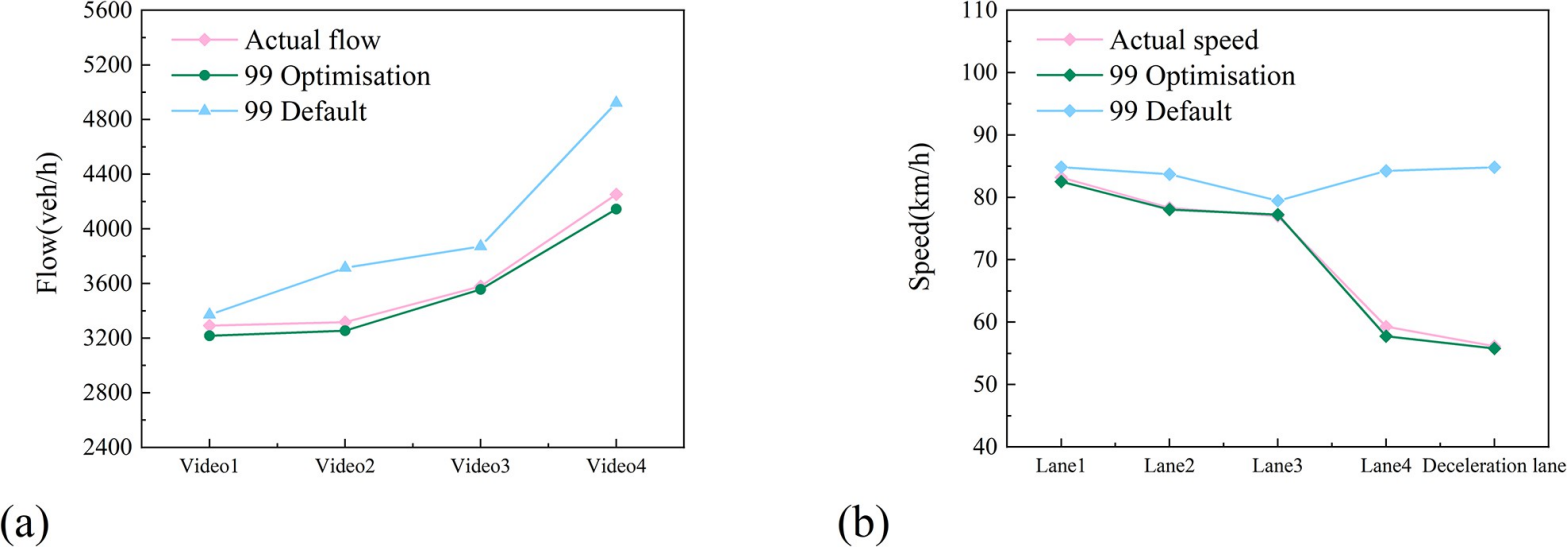
Comparison of Simulation Data and Measured Data: (a) Flow; (b) Speed.

Upon integration of the measured data, a comparative analysis was conducted between the optimised Wiedemann99 model, calibrated with inputted measured data, and its default counterpart. The comparison is shown in Fig 7. The optimised model exhibited errors in actual flow and velocity of less than 3% and 4%, respectively, significantly outperforming the default Wiedemann99 simulation model. This underscores its effectiveness in accurately capturing the operational intricacies of vehicles within the short-distance continuous diversion area, thereby establishing a robust foundation for further studies on the area’s actual traffic capacity.

### 4.3 Analysis of main influencing factors

#### 4.3.1 Influencing factor selection

According to American HCM 2010, the factors influencing capacity primarily include road conditions (such as longitudinal slope gradient and lane width), traffic conditions (vehicle composition and lane distribution), and technical conditions (primarily intelligent transportation systems). The analysis of district characteristics indicates that mountainous urban short-distance continuous diversion areas differ significantly from typical diversion areas due to terrain constraints. Moreover, the necessity for vehicles to frequently change lanes quickly and over short distances complicates traffic operations. Consequently, six geometric parameters were identified as influential: diversion-point spacing, deceleration lane length, number of mainline lanes, lane width, ramp gradient, and ramp curvature; along with two traffic parameters: primary and secondary diversion ratios.

Experiments were conducted using a traffic simulation model (VISSIM), and the single-variable control method was utilized to determine the passing capacity values under different combinations of factors. Given the extensive range of feature sequences, each influencing factor was categorised into three variable groups, yielding 38 data sets through simulation experiments. Table 6 lists some of the data.

**Table 6:** Capacity values for different combinations of factors.

Diversion-point spacing (m)	Length of deceleration lanes (m)	Mainline (of communication) lane number	Lane width (m)	Ramp Gradient	ramp curvature	primary diversion ratio	Secondary diversion ratio	Capacity (veh/h)
10	100	3	2.75	3.0	1.0	0.2	0.1	3515
10	100	3	2.75	3.0	1.0	0.2	0.3	3565
10	100	3	2.75	3.0	1.0	0.2	0.5	3615
…	…	…	…	…	…	…	…	…
10	100	3	2.75	3.0	1.0	1.0	0.1	2108
10	100	3	2.75	3.0	1.0	1.0	0.3	2214
10	100	3	2.75	3.0	1.0	1.0	0.5	3239
…	…	…	…	…	…	…	…	…

**Fig 8:**
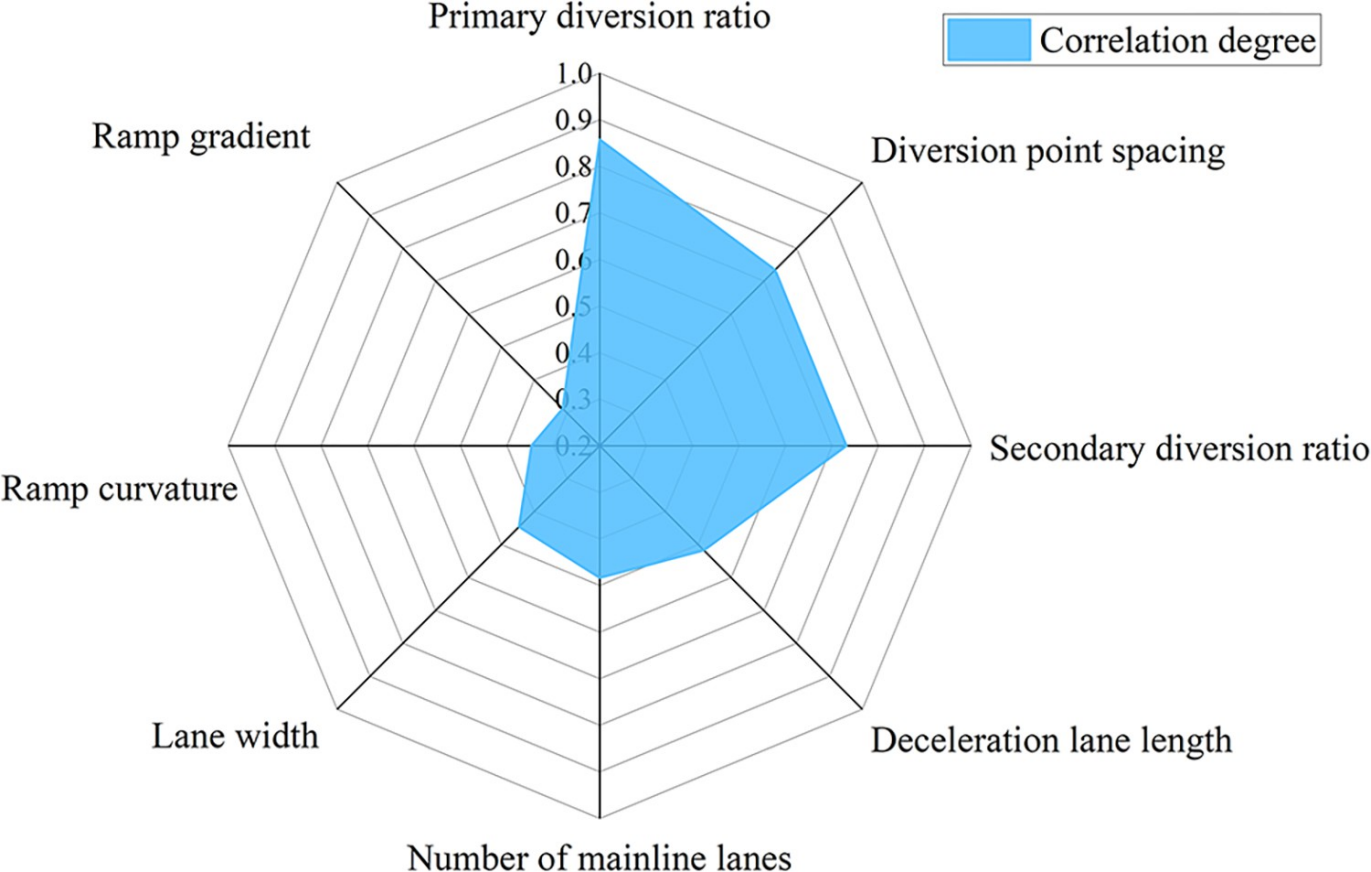
Results of correlation analysis.

Based on the grey system correlation degree theory, considering the capacity of the diversion area as a reference sequence, we studied the degree of influence of the changeability trend of the diversion-point spacing, deceleration lane length, and other factors on the road capacity. Combined with the data shown in Table 7, the correlation degree of each influencing factor and the traffic capacity of the diversion area was calculated, and the results are shown in Fig 8.

**Table 7:** Capacity values for different combinations of factors.

Serial number	Diversion-point spacing (m)	Primary diversion ratio	Secondary diversion ratio	Capacity (veh/h)
1	10	0.0	0.0	3368
2	10	0.0	0.1	3368
3	10	0.0	0.2	3368
4	10	0.0	0.3	3368
5	10	0.0	0.4	3368
6	10	0.0	0.5	3368
…	…	…	…	…
430	120	1.0	0.3	2970
431	120	1.0	0.4	3503
432	120	1.0	0.5	3500

The primary diversion ratio has a strong influence on the capacity; the diversion-point spacing and secondary diversion ratio have a stronger influence on the capacity; the length of the deceleration lanes, number of mainline lanes, and lane width have a moderate influence; and the curvature of the ramps and ramp gradient has a weaker influence. As a result, three parameters, namely, the diversion-point spacing, primary diversion ratio, and secondary diversion ratio were selected as the main influencing factors, and the capacity calculation model was constructed based on these factors.

**Fig 9:**
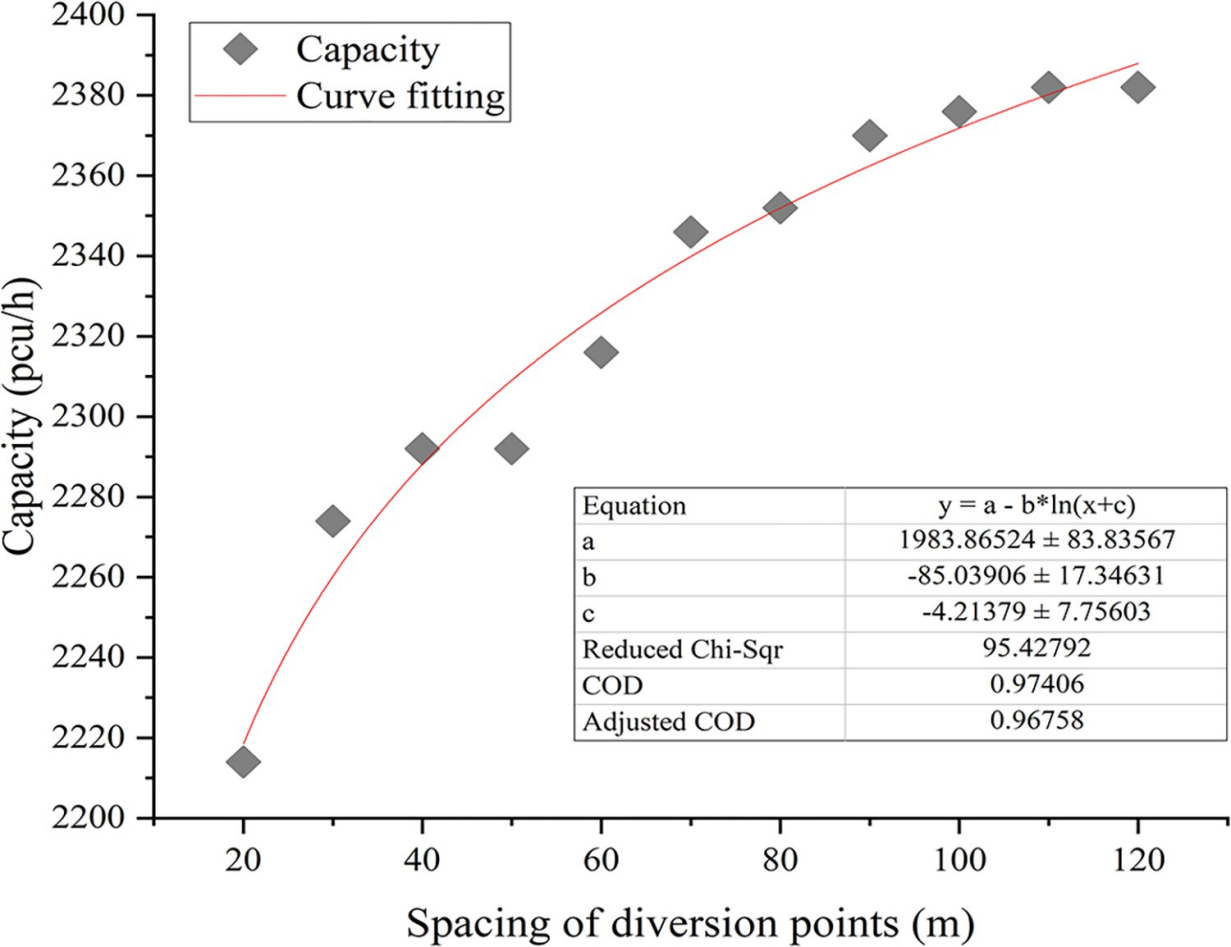
Simulation results of diversion-point spacing.

**Fig 10:**
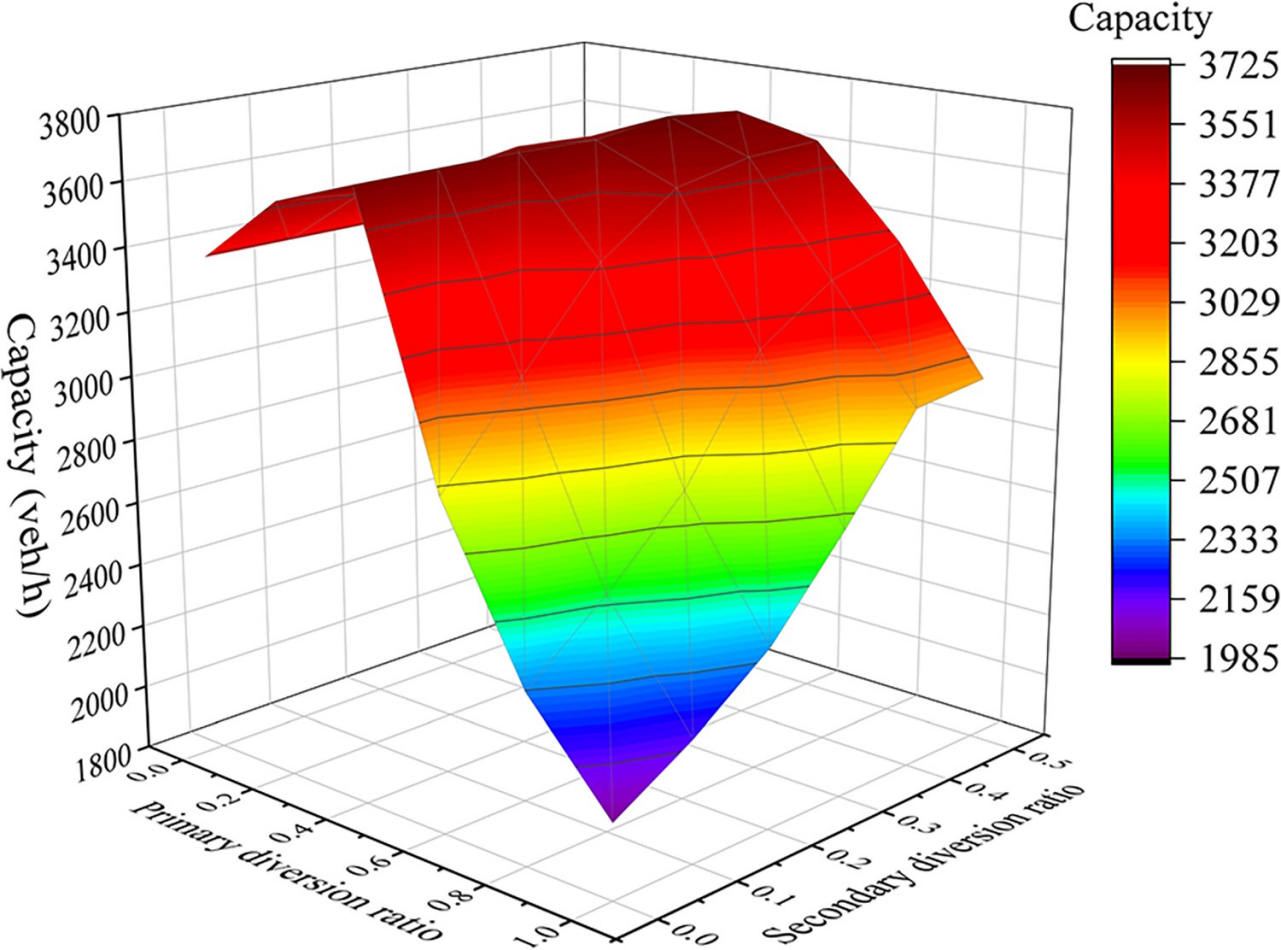
Throughput capacity values.

1. *Diversion-point spacing*. Differences in throughput capacity are evident across varying distances between diversion points, with throughput capacity fluctuating in accordance with changes in diversion point spacing. The experimental throughput capacity values are depicted as a scatter plot against diversion-point spacing, as illustrated in Fig 9. Notably, within the interval of diversion-point spacing ranging from 10–80 m, the throughput capacity exhibits substantial increments, highlighting the pronounced impact of diversion-point spacing within range on the throughput capacity of the diversion area.
2. *Diversion ratio*. Two diversion points were situated within a short-distance continuous diversion area. This paper analyzed the influence of varying combinations of primary and secondary diversion ratios on the throughput capacity of the diversion area. The experimental throughput capacity values are depicted in a three-dimensional surface plot against diversion ratios, as shown in Fig 10. In the range [0, 0.5], as the primary diversion ratio increased, the throughput capacity of the diversion area gradually increased to its peak of 3,720 vehicles per hour (veh/h). However, within the interval [0.5, 1.0], the throughput capacity decreased as the primary diversion ratio changed. This highlights the pronounced effect of the primary diversion ratio on the diversion area’s throughput capacity. Moreover, when the primary diversion ratio was held constant within the interval [0, 0.5], the throughput capacity of the diversion area remained relatively stable and exhibited no discernible correlation with the secondary diversion ratio. Conversely, within the primary diversion ratio range [0.5, 1.0], the secondary diversion ratio exerted a significant influence on the throughput capacity of the diversion area.

#### 4.3.2 Factor combination analysis

Simulation experiments were conducted on the three main influencing factors, namely, diversion-point spacing, primary diversion ratio, and secondary diversion ratio, to extract the capacity under different combinations of influencing factors. A total of 432 sets of experimental data were obtained, some of which are shown in 7.

**Fig 11:**
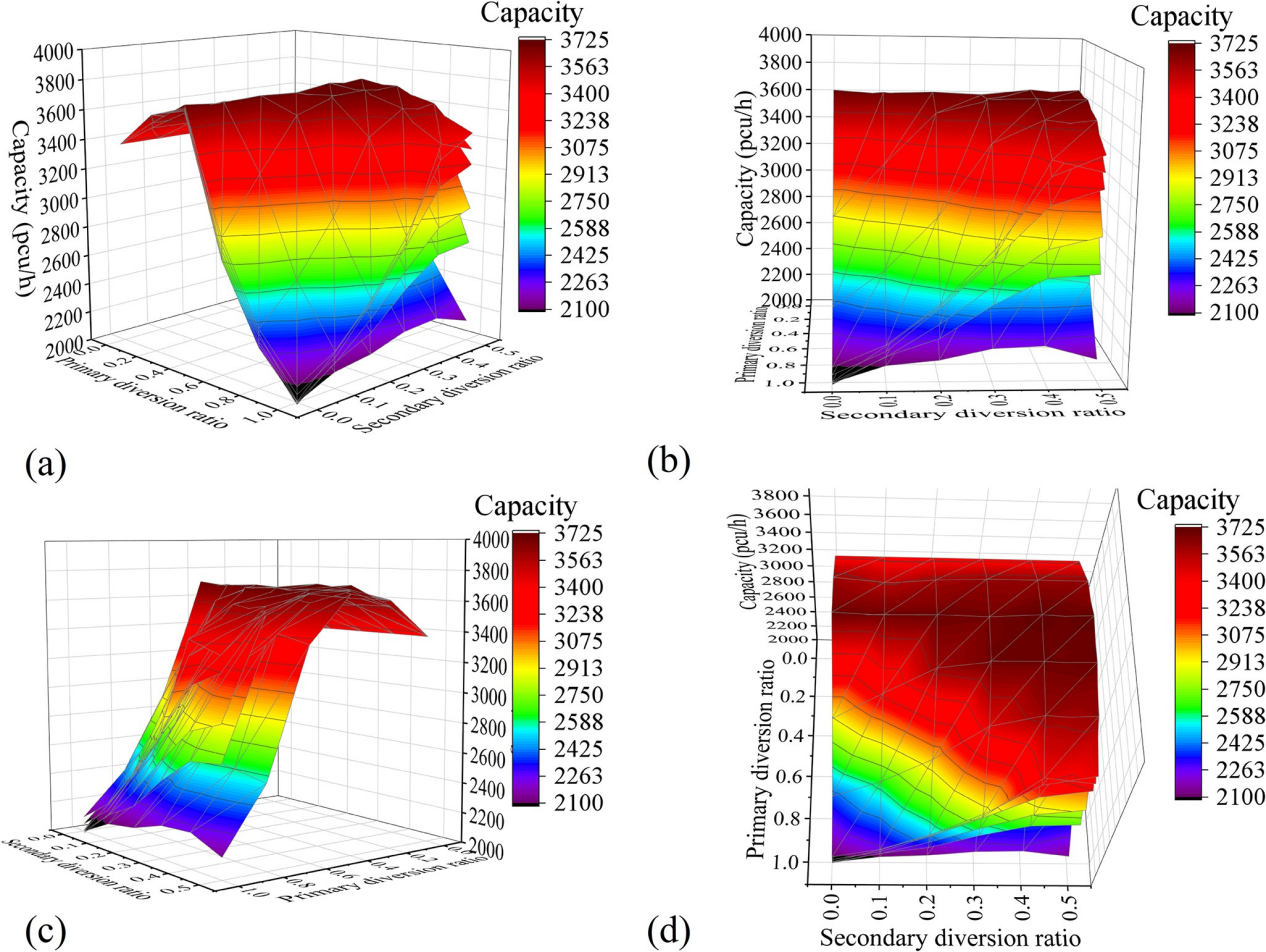
Experimental results of the combination of the main influencing factors: (a) Viewpoint 1; (b) Viewpoint 2; (c) Viewpoint 3; and (d) Viewpoint 4.

**Fig 12:**
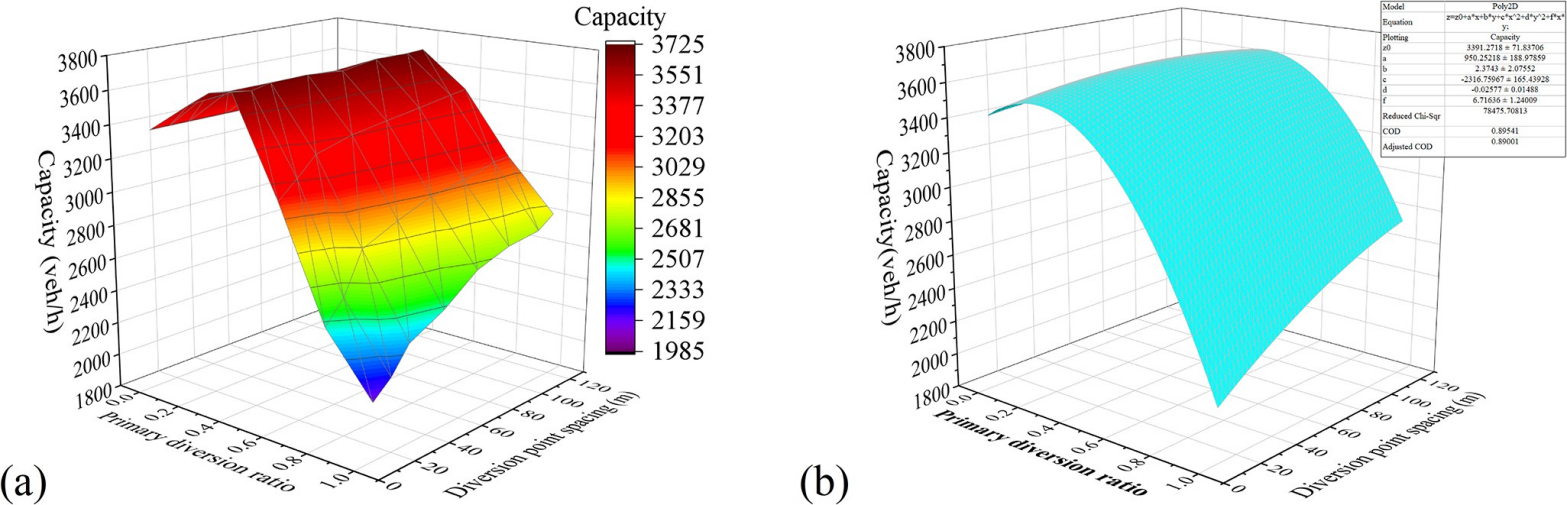
Experimental results of the combination of the primary diversion ratio and diversion-point spacing: (a) Capacity Surface Map; and (b) Fitting surface diagrams.

**Fig 13:**
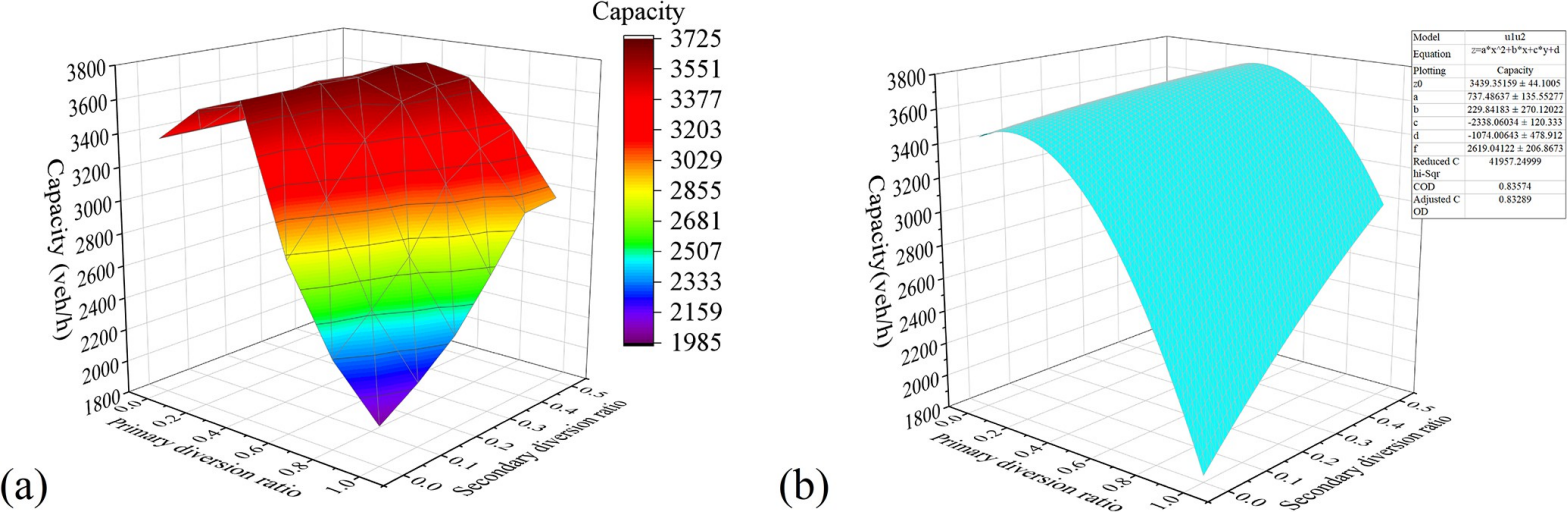
Experimental results of the combination of the primary and secondary diversion ratios: (a) Capacity Surface Map; and (b) Fitting surface diagrams.

**Fig 14:**
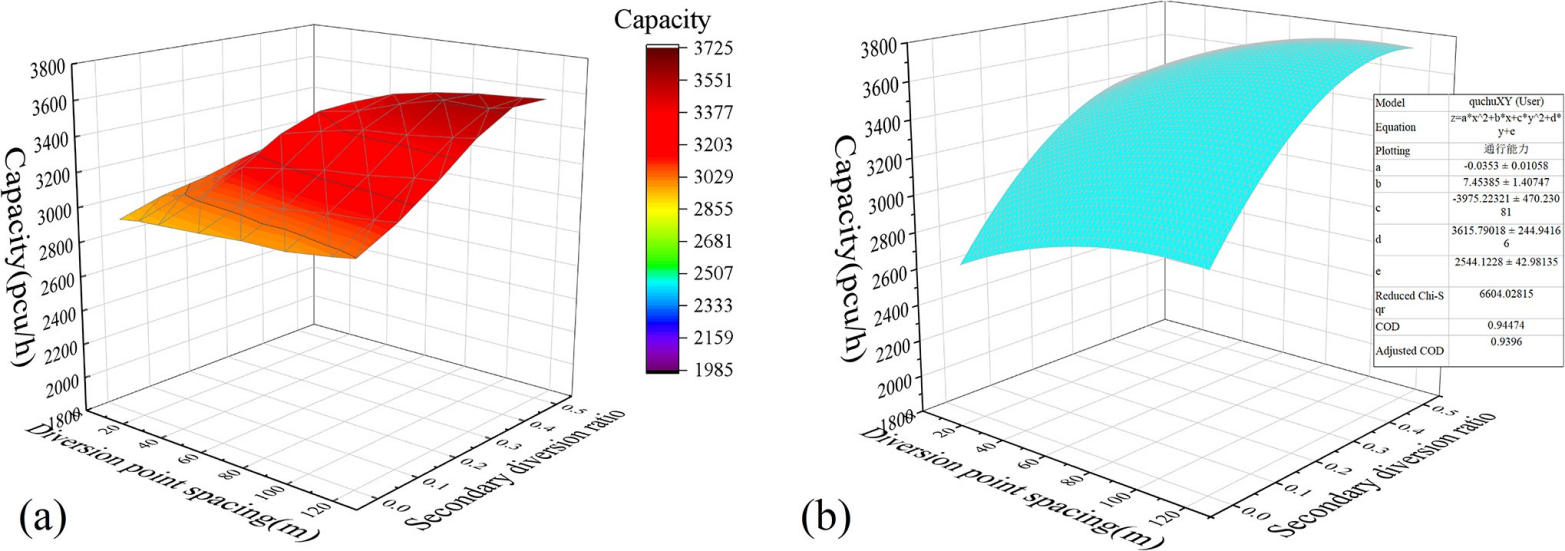
Experimental results of the combination of the diversion-point spacing and secondary diversion ratio: (a) Capacity Surface Map; and (b) Fitting surface diagrams.

1. *Multifactor combination analysis*. Fig 11 shows the surface plot of the relationship between the influencing factors and the capacity drawn with the simulation experimental data. The X-axis and Y-axis coordinates of the graph are the primary and secondary diversion ratios, respectively. Each surface layer corresponds to a different spacing of diversion points, the bottom to the top surface layer corresponds to 10–120 m, and the step length of the surface is set to 20 m for convenience of observation.
2. *Two-factor surface-fitting analysis*. To determine the relationship between each influencing factor and the capacity, a surface plot of the relationship between dual influencing factors and capacity was plotted and fitted using a function. The optimal fitting results are as shown in Figs 12–14. According to the fitting results of the simulation data, *μ*_1_ and *L* have a quadratic relationship with the capacity *C*, and *μ*_2_ has a linear relationship with *C* at the highest goodness-of-fit.

From the figure, the primary diversion ratio *μ*_1_ has the greatest influence on the capacity *C*. For the secondary diversion ratio *μ*_2_, the diversion-point spacing *L* is fixed. With the increase of *μ*_1_, *C* shows the trend of increasing and then decreasing, and it reaches its maximum value at *μ*_1_ = 0.4. Hence, the relationship between *μ*_1_ and *C* is a first-order quadratic polynomial.

For values [0, 0.5], *μ*_2_ and *L* have no significant effect on *C*. Therefore, considering the establishment of the segmented function, when *μ*_1_ is in the range [0, 0.5], only the relationship between *μ*_1_ and *C* should be investigated. When the value is (0.5, 1], with the increase of *μ*_2_, the growth rate of *C* is faster and then slower. Hence, the relationship between *μ*_2_ and *C* may be a linear, logarithmic, or quadratic polynomial. *L* is similar to *μ*_2_, and the growth rate of neighbouring surfaces gradually slows from the bottom to the top. Hence, the relationship between *L* and *C/* may be consistent with the relationship of a logarithmic or quadratic polynomial. The exact functional form can be determined via surface fitting.

### 4.4 Proposed model and validation

#### 4.4.1 Proposed capacity model

The above experimental analysis shows that the capacity of the diversion area *C* has a significant quadratic polynomial correlation with the primary diversion ratio *μ*_1_ and the spacing of the diversion points *L* and that *C* has a linear correlation with the secondary diversion ratio *μ*_2_. Therefore, the following hypotheses are made for the relationship between *C*, *L*, *μ*_1_, and *μ*_2_ in the form of a surface-fitting function:

$$C=\{\begin{array}{l} a\mu_{1}^{2}+b\mu_{1}+c\;\mu_{1}\in [0,0.5]\\ dL^{2}+e\mu_{1}^{2}+fL\mu_{1}+gL\mu_{2}+h\mu_{1}+i\mu_{2}+j\;\mu_{1}\in (0.5,1] \end{array}$$ (4)

where *C* represents the actual capacity of the diversion area in veh/h, *L* represents the distance between diversion points in the range [10 m, 120 m], *μ*_1_ represents the primary diversion ratio in the range [0, 1], *μ*_2_ represents the secondary diversion ratio in the range [0, 0.5], and *a*, *b*, *c*, *d*, *e*, *f*, *g*, *h*, *i*, *j* represent the regression coefficients.

The data obtained through the combination of the VISSIM simulation model experiments in the model were substituted into the recommended formula for the passage capacity for multiple regression analysis. The regression coefficients of the model are shown in Table 8.

**Table 8:** Model regression coefficients and confidence intervals.

Regression coefficient	Estimated value	Standard error	Standard Error Test	Confidence interval (math.)	
				95% lower confidence limit	95% confidence limit
a	-1476.785714	101.0550265	6.84%	-1676.818	-1276.753
b	1381.785714	42.07256255	3.04%	1298.506	1465.066
c	3368.00	3.300443345	0.10%	3361.467	3374.533
d	-0.048258258	0.01222527	25.33%	-0.073	-0.024
e	1837.916667	528.5622973	28.76%	786.628	2889.206
f	6.547823057	1.532511783	23.40%	3.500	9.596
g	23.4676954	2.451916752	10.45%	18.591	28.344
h	-5474.735371	850.6990552	15.54%	-7166.742	-3782.729
i	1036.342596	130.922919	12.63%	775.942	1296.743
j	5422.682982	330.0963481	6.09%	4766.135	6079.231

The formula for calculating the capacity derived from the fitting in this study is shown in Eq 5:

$$C=\{\begin{array}{l} -1476.79\mu_{1}^{2}+1381.79\mu_{1}+3368\;\mu_{1}\in [0,0.5]\\ -0.05L^{2}+1837.92\mu_{1}^{2}+6.55L\mu_{1}+23.47L\mu_{2}-5474.74\mu_{1}+1036.34\mu_{2}+5422.68\;\mu_{1}\in (0.5,1] \end{array}$$ (5)

where *C* represents the actual capacity of the diversion area in veh/h, *L* represents the distance between diversion points in the range [10 m, 120 m], *μ*_1_ represents the primary diversion ratio in the range [0, 1], and *μ*_2_ represents the secondary diversion ratio in the range [0, 0.5].

#### 4.4.2 Model validation

**Fig 15:**
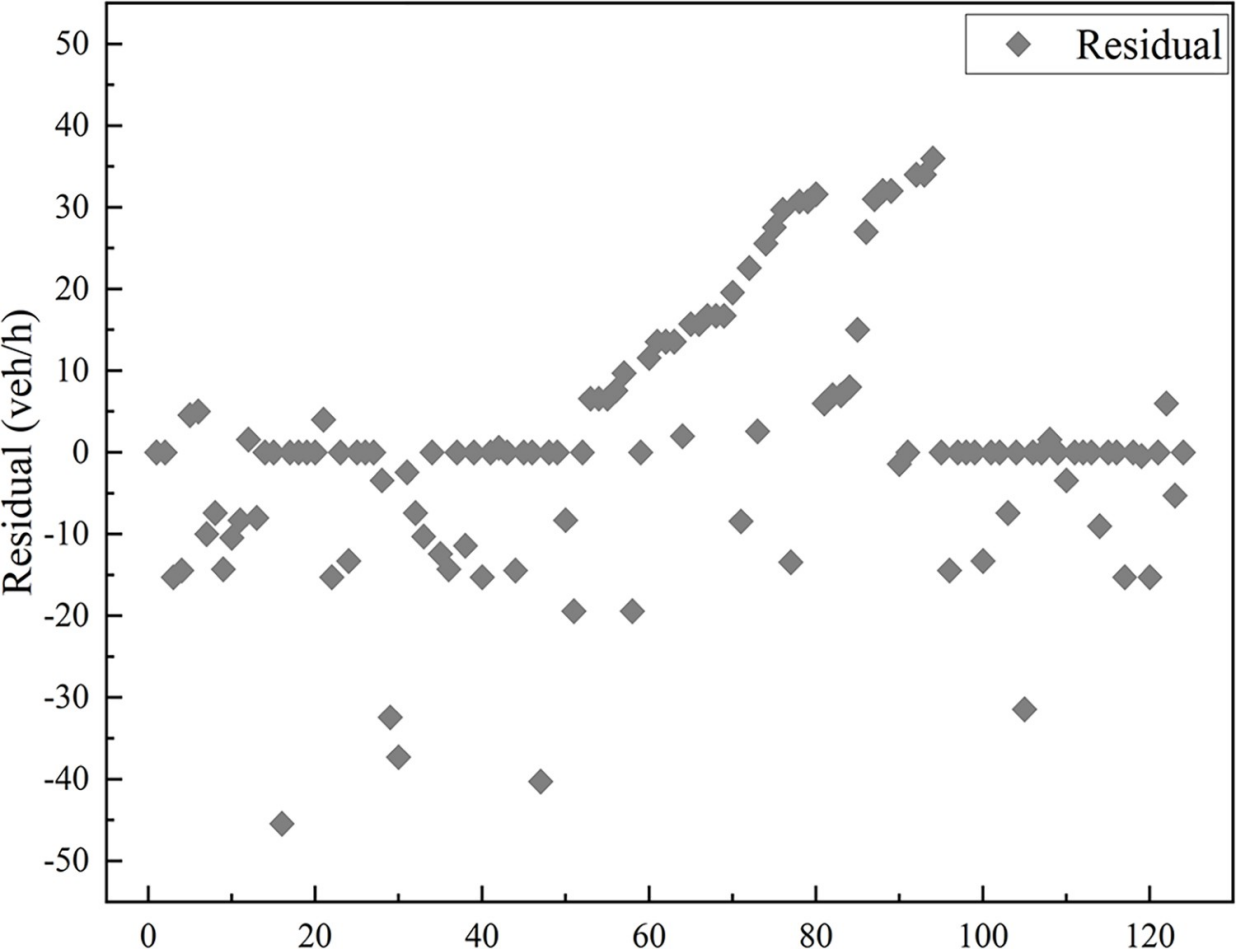
Scatterplot of residual distribution.

**Fig 16:**
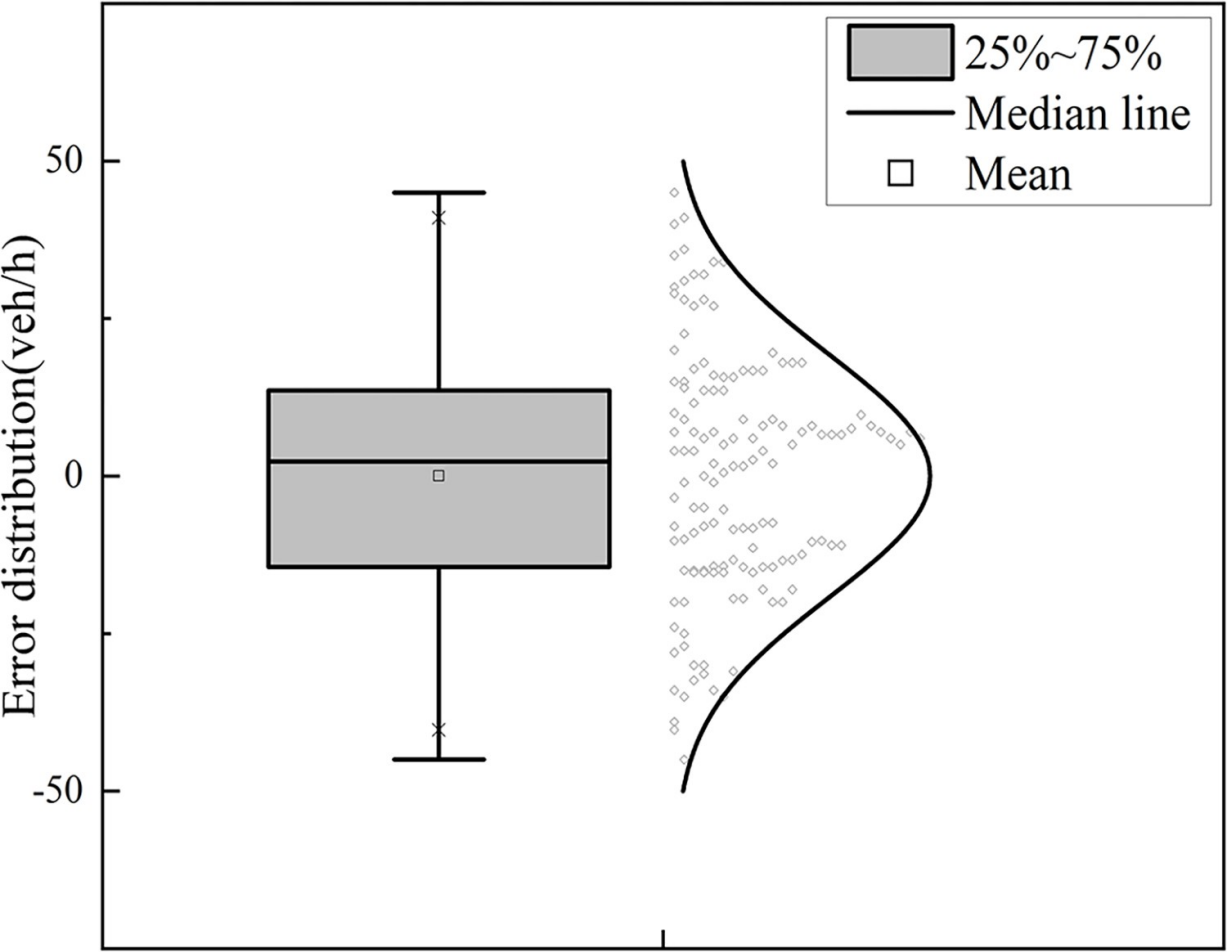
Box line plot of residual distribution.

**Fig 17:**
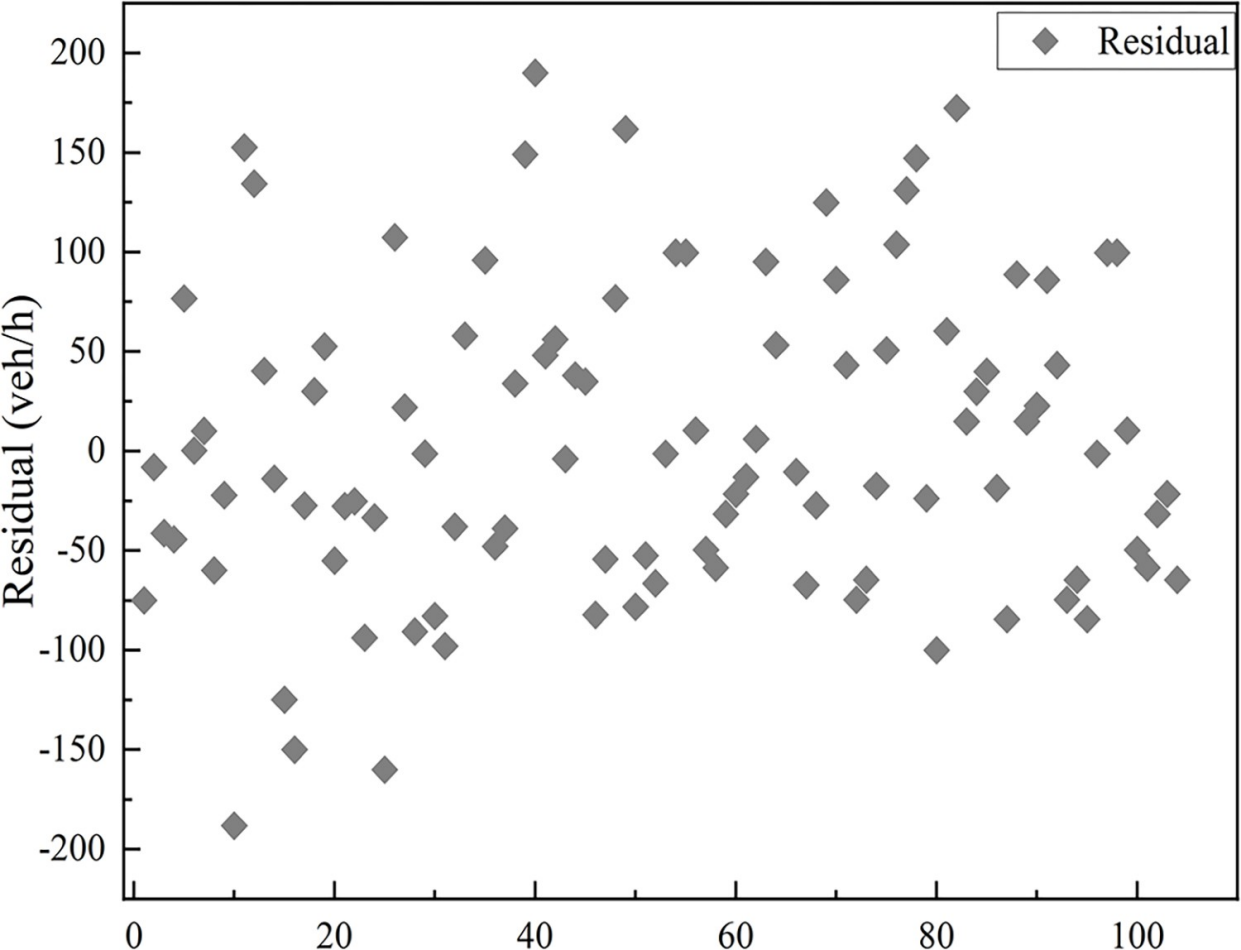
Scatterplot of residual distribution.

**Fig 18:**
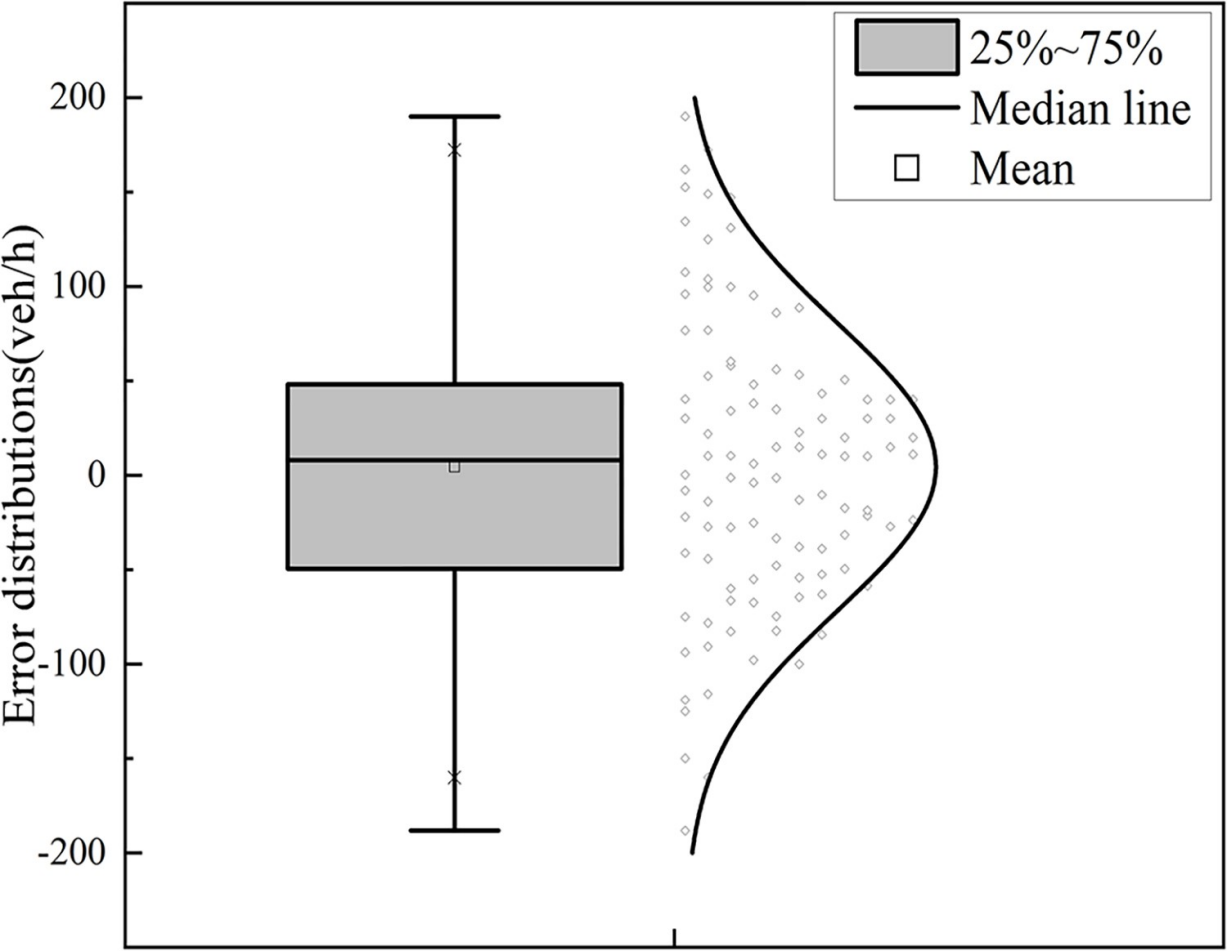
Box line plot of residual distribution.

1. *Confidence interval analysis of regression coefficients*. The confidence intervals of all regression coefficients are free of zeros, and the effects of the independent variables on the model parameters are monotonic with good model stability.
2. *Goodness-of-fit test*. Under the conditions of a 95% confidence level and sample size of n = 432, when *μ*_1_ takes on a value in [0, 0.5], the value of *R* is 0.987, and the value of *R*^2^ is 0.9. When *μ*_1_ takes on a value in (0.5, 1], the value of *R* is 0.969, the value of *R*^2^ is 0.96, and the model fit is good.
3. *Fitting accuracy test*.

1) When *μ*_1_ assumes a value in the range [0, 0.5], the corresponding residual distribution is depicted in Figs 15 and 16. The residuals calculated by the model are distributed within the interval [-50 veh/h, 50 veh/h] with a mean value of 0.03 veh/h, value of 2.29 veh/h, and maximum error of no more than 2% of the total capacity.

2) When *μ*_1_ assumes a value in the range (0.5, 1], the corresponding residual distribution is depicted in Figs 17 and 18. The residuals of the capacity calculated by the model are distributed in the range of [-200 veh/h, 200 veh/h] with a mean value of 4.46 veh/h, a median value of 8.03 veh/h, and a maximum error of no more than 6% of the total capacity. The accuracy of the model is high.

### 4.5 Field survey declaration

According to the "Interim Regulations on the Flight Management of Unmanned Aerial Vehicles" in China, micro unmanned aerial vehicles (UAVs) operating below 120 meters in designated airspace do not require a pilot’s license or flight application. In this study, we employed the DJI MINI 2 drone, which weighs 249 grams and qualifies as a micro UAV. Therefore, no special licensing or applications were required for its operation during filming. Throughout this research, we ensured compliance with relevant Chinese regulations and policies, particularly for aerial photography. We thoroughly reviewed flight regulations and implemented measures to ensure safety and legality. According to current Chinese policy, no specific licensing requirements were necessary for on-site visits. However, we strictly adhered to urban flight regulations and took precautions to minimize disturbances and safety risks. All research activities were conducted in accordance with Chinese regulations.

## 5. Results and discussion

### 5.1 Calculating capacity results

Using the northern side of the Huacun Interchange diverging area as a case study, vehicle trajectory data were collected under both free-flow and congested conditions through six sets of aerial video recordings. Parameters, including speed and parking spacing, were calibrated based on empirical data and theoretical models. Table 9 presents the determined ranges and optimal values for each parameter.

**Table 9:** Key influencing parameters of the simulation model.

Parameters	Meaning of Parameters	Default Value	Recommended Value Range	Determining Parameters
CC0	Parking Spacing	1.5	0.5–2.5	1.82
CC1	Time Headway	0.9	0.7–1.7	1.37
CC2	Following Vehicle Variables	4	2–8	2.71
CC4	Threshold of Negative Following State	-0.35	0.05–1.05	-0.95
CC5	Threshold of Positive Following State	0.35	0.7–1.11	0.95
Safety Distance Reduction Coefficient		0.6	0.1–0.6	0.2

Concurrently, diversion ratios for both primary and secondary flows during peak hours were derived from the videos gathered, with the spacing between diversion points fixed at 70 m. Utilising the calibrated simulation model, a series of experiments combining multiple parameters were performed to ascertain the true traffic capacity of the short-distance continuous diversion area at Huacun Interchange, as illustrated in Table 10.

**Table 10:** Actual capacity for different combinations of factors.

Serial number	Diversion-point spacing (m)	Primary diversion ratio	Secondary diversion ratio	Actual capacity (veh/h)	Calculated capacity (veh/h)
1	70	0.84	0.23	2816	2877
2	70	0.79	0.25	3055	3032
3	70	0.53	0.37	3861	4027
4	70	0.62	0.31	3677	3605
5	70	0.81	0.28	3102	3071
6	70	0.70	0.33	3511	3451

### 5.2 Comparison between calculated and actual capacity

**Fig 19:**
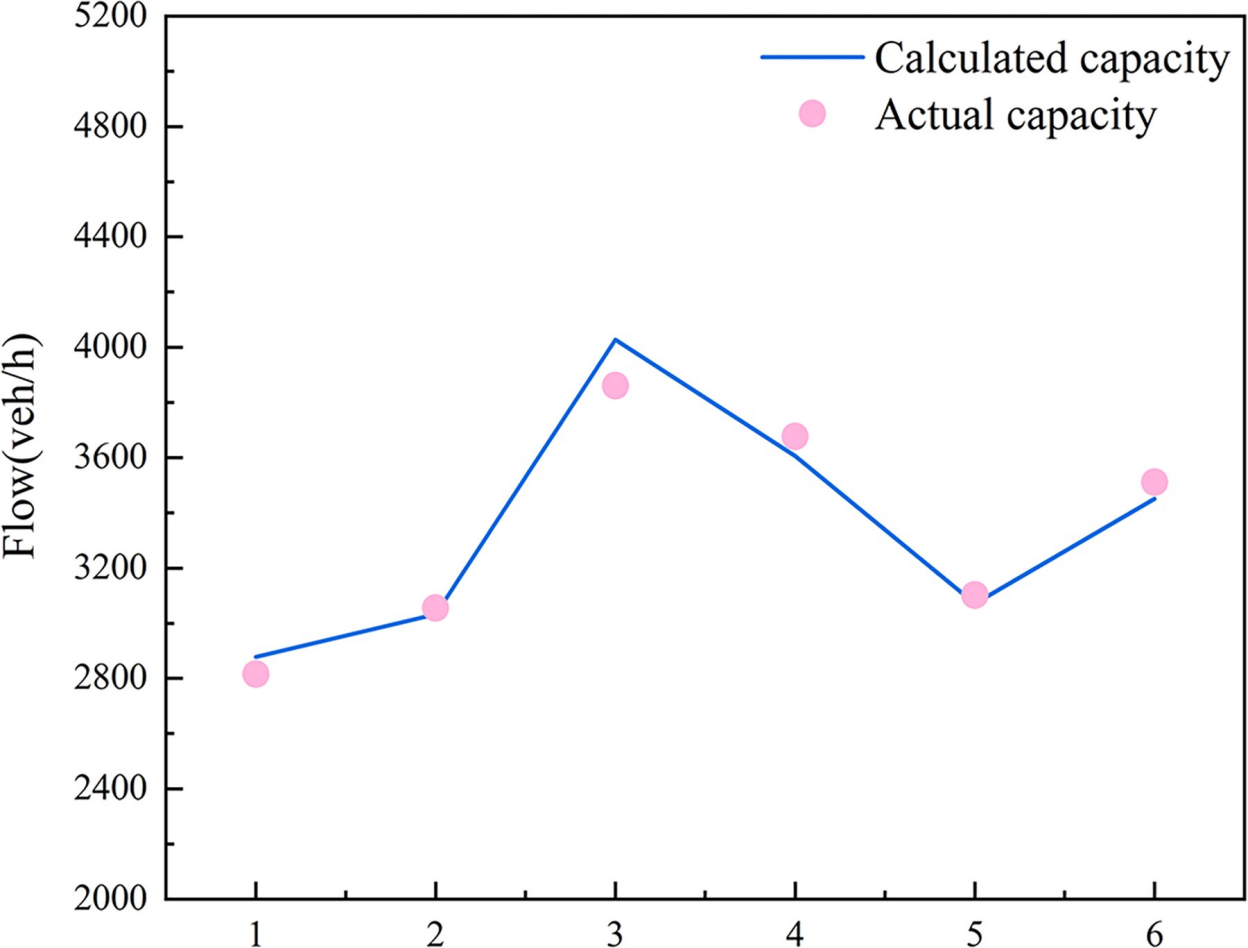
Comparison of calculated and actual capacities in diversion areas.

The actual traffic capacity values obtained from simulation experiments were compared with those calculated using the traffic capacity model, the results are presented in Table 11 and Fig 19. The difference between the two sets of values is minimal, with an error percentage ranging from 0.77%–4.11%. This suggests that the calculation model demonstrates good predictive ability and consistency across various parameter combinations. Notably, the calculated value for Combination 3 slightly exceeds the actual value, showing a 4.11% error. This indicates a tendency for overestimation in specific cases, highlighting the need to adjust weights for certain parameter combinations.

**Table 11:** Comparison of traffic capacity values.

Serial number	Diversion-point spacing (m)	Primary diversion ratio	Secondary diversion ratio	Actual capacity (veh/h)	Calculated Traffic Capacity (veh/h)	Error
1	70	0.84	0.23	2816	2877	2.12%
2	70	0.79	0.25	3055	3032	0.77%
3	70	0.53	0.37	3861	4027	4.11%
4	70	0.62	0.31	3677	3605	2.01%
5	70	0.81	0.28	3102	3071	1.02%
6	70	0.70	0.33	3511	3451	1.74%

The current traffic capacity calculation model generally performs well, with errors compared to the actual values within an acceptable range of 5%. This demonstrates its high predictive ability and consistency. However, further optimization and adjustment of model parameters, particularly to address overestimation in specific cases, can enhance the model’s accuracy and reliability. This will provide more scientific and precise data support for traffic planning and management in complex scenarios.

### 5.3 Practical implications of this study

The traffic capacity calculation model evaluates the capacity and efficiency of various roads and sections [31]. It produces results that identify traffic bottlenecks and sections in need of expansion or improvement, providing scientific and accurate data to support the development of feasible traffic planning and infrastructure construction plans [32]. Furthermore, the model serves to verify the effectiveness of different traffic measures. By simulating diverse traffic signal settings, traffic accidents, emergencies [33], and vehicle density and flow speed conditions across areas, it pinpoints the causes of traffic congestion and aids traffic management departments in devising targeted measures to alleviate such congestion.

Amid significant advances in vehicle-road coordination in recent years, the traffic capacity calculation model also supplies essential data for vehicle-road coordination systems [34], enhancing communication between vehicles and road infrastructure [35]. For example, by specifying certain vehicle travel paths or speeds, the model reduces conflicts and queue lengths at divergences, thereby ensuring a more stable flow of traffic in vehicle-dense zones [36].

Beyond practical applications, the findings from the traffic capacity calculation model provide a scientific foundation for the formulation of government policies. These include setting investment priorities, establishing traffic emission reduction targets, and developing long- and short-term strategic plans [37].

## 6. Conclusion

Short-distance continuous diversion areas on expressways in mountainous cities connect major transportation hubs and key urban regions, promoting intercity connectivity and development while enhancing overall traffic operation efficiency. This study established a traffic capacity calculation model by considering the characteristics of diversion areas and incorporating the results from influencing factor analysis. Based on the analysis of field survey data, the unique road and traffic characteristics of short-distance continuous diversion areas were examined. A multifactor combination analysis was conducted to comprehensively assess the impact of various factors on traffic capacity, making the model more realistic. The constructed traffic capacity calculation model employed theoretical analytical methods to accurately calculate the capacity of diverging areas. Validation showed that the error between the calculated and actual traffic capacity was less than 5%, indicating high model accuracy. The research findings effectively support traffic planning and management in diversion areas and lay a foundation for traffic operation management in complex diverging areas under vehicle-road coordination environments.

This study constructed a calculation model for traffic capacity based on three main influencing factors: diversion-point spacing, primary diversion ratio, and secondary diversion ratio. However, vehicle behaviours, such as deceleration and frequent lane changes in secondary diversion areas, also led to sudden decreases in traffic capacity. Future research should consider these parameters when constructing traffic capacity calculation models to improve the model. To further enhance the model’s accuracy, subsequent research can introduce more actual data and multivariate analysis to finely calibrate and optimise model parameters, thereby enhancing the model’s adaptability and accuracy to better support planning and management in complex traffic environments. Additionally, due to the difficulty in obtaining actual survey data, this study only considered the "right-side ramp continuous diversion" setup form. Future research needs to study other setup forms of diversion areas such as "mainline right-side continuous diversion" and "mainline right-then-left diversion".

## Supporting information

S1 File It contains all the data files for this manuscript. (XLSX)
